# Psychometric network models from time-series and panel data

**DOI:** 10.1007/s11336-020-09697-3

**Published:** 2020-03-11

**Authors:** Sacha Epskamp

**Affiliations:** Department of Psychology: Psychological Methods Groups, University of Amsterdam, PO Box 15906, 1001 NK Amsterdam, The Netherlands

**Keywords:** network psychometrics, Gaussian graphical model, structural equation modeling, dynamics, time-series data, panel data

## Abstract

Researchers in the field of network psychometrics often focus on the estimation of Gaussian graphical models (GGMs)—an undirected network model of partial correlations—between observed variables of cross-sectional data or single-subject time-series data. This assumes that all variables are measured without measurement error, which may be implausible. In addition, cross-sectional data cannot distinguish between within-subject and between-subject effects. This paper provides a general framework that extends GGM modeling with latent variables, including relationships over time. These relationships can be estimated from time-series data or panel data featuring at least three waves of measurement. The model takes the form of a graphical vector-autoregression model between latent variables and is termed the *ts-lvgvar* when estimated from time-series data and the *panel-lvgvar* when estimated from panel data. These methods have been implemented in the software package *psychonetrics*, which is exemplified in two empirical examples, one using time-series data and one using panel data, and evaluated in two large-scale simulation studies. The paper concludes with a discussion on ergodicity and generalizability. Although within-subject effects may in principle be separated from between-subject effects, the interpretation of these results rests on the intensity and the time interval of measurement and on the plausibility of the assumption of stationarity.

## Introduction

Researchers in the field of *network psychometrics* (Marsman et al., 2018) study the estimation of multivariate statistical models in attempts to map out the complex interplay of interactions between variables. This field emerged from the *network perspective* on psychology (Borsboom, 2017; Cramer, Waldorp, van der Maas, & Borsboom, 2010)—departing from the latent variable model and instead conceptualizing observed variables (e.g., attitudes, symptoms, and moods) as causal agents in a complex interplay of psychological (and other) components. In recent years, network models have become useful additions to the psychometric toolbox. For example, network models provide useful visualization tools to check the fit of latent variable models (Epskamp, Cramer, Waldrop, Schmittmann, & Borsboom, 2012), are closely tied and often equivalent to latent variable models (Marsman et al., 2018), are often uniquely identified (Epskamp, Waldorp, Mõttus, & Borsboom, 2018), and may provide exploratory insight into the underlying factor structure by investigating its clustering (Golino & Epskamp, 2017).

In a psychometric network model,^1^ variables are represented by *nodes* that are connected by *edges*, which are weighted according to some statistic. In this paper, I focus on two particular network models now routinely used in the analysis of continuous data: the Gaussian graphical model (GGM; Epskamp, Waldorp, et al., 2018; Lauritzen, 1996) and the graphical vector-autoregression model (GVAR; Epskamp, Waldorp, et al., 2018; Wild et al., 2010). The GGM forms an undirected network model, in which edges represent partial correlation coefficients. The GGM is closely tied, but not fully equivalent, to the directed structures typically used in structural equation modeling (SEM; Epskamp, Rhemtulla, & Borsboom, 2017) and is often estimated from cross-sectional data (Fried et al., 2017), in which many subjects are measured only once. The (lag-1) GVAR model takes the form of a generalization of the GGM in single-subject time series (Epskamp, Waldorp, et al., 2018). In the GVAR, temporal dependencies are modeled via a regression on the previous measurement occasion, which leads to a matrix of regression coefficients that can also be used to draw a directed network model (Bringmann et al., 2013)—often termed the *temporal network* because it encodes predictive effects over time. The remaining variances and covariances (i.e., the covariance structure after controlling for the previous measurement occasion) can be modeled as a GGM, which is also termed the *contemporaneous network*. When time series of multiple subjects are available, a third GGM can be formed on the between-subject effects (relationships between stable means)—also termed the *between-subject* network.

Current practices in network psychometrics feature two pressing limitations. (1) Although network modeling has been proposed as an alternative to latent variable modeling (i.e., covariation is caused by one or more unobserved common causes), the complete omission of latent variables may be one step too far (Bringmann & Eronen, 2018; Fried & Cramer, 2017; Guyon, Falissard, & Kop, 2017). Network models for observed variables rely on the assumption that all causally interacting variables are observed without error. However, a certain level of measurement error (Schuurman, Houtveen, & Hamaker, 2015) should be assumed in psychological data. (2) Network models are now often estimated from cross-sectional data. However, such results are not reflective of within-subject dynamics over time (Bos et al., 2017; Molenaar, 2004). Cross-sectional analysis cannot distinguish between within- and between-subject variances (Hamaker, 2012) or indeed investigate temporal effects over time. In principle, panel data, in which many subjects are measured a few times, can be used to distinguish between within- and between-subject effects (Hamaker, Kuiper, & Grasman, 2015), but have only been sparingly discussed in network psychometrics (e.g., Rhemtulla, Van Bork, & Cramer, 2017). Prior literature has tackled these topics separately. For example, Epskamp, Rhemtulla, and Borsboom (2017) addressed Limitation 1 by proposing to form a psychometric framework to incorporate GGMs in latent variable models, both at the latent level (latent network models) and at the residual level (residual network models). The multi-level GVAR model (Epskamp, Waldorp, et al., 2018) can overcome Limitation 2 but does not include latent variables and requires intensive repeated measures of many subjects.

The goal in this paper is to combine the above-described solutions into a general framework. The framework emerges by combining a general factor model and a GVAR model and is referred to as the *lvgvar* model.^2^ I will discuss estimation in two very distinct settings:

- Time-series data of a single subject. This setting will be termed *ts-lvgvar*.
- Panel data of many subjects measured on a few (at least three) measurement occasions. This setting will be termed *panel-lvgvar*.

These settings differ crucially from one another. *ts-lvgvar* concerns a fixed subject (fixed subject *p*), which involves variation over measurement occasions (random time *T*). Meanwhile, *panel-lvgvar* concerns variation over subjects (random subject *P*) at a few fixed time points $t, t+1, t+2, \ldots$. The *panel-lvgvar* is a multi-level model with random effects on the mean structure. I showcase both methods using two empirical examples and assess the performance of model search strategies through two large-scale simulation studies. Finally, I discuss the generalizability of results between these settings in detail. The *lvgvar* framework is implemented in the open-source software package *psychonetrics*.^3^

## Preliminary Topics

### Notation

This paper uses a similar notation as Epskamp, Waldorp, et al. (2018). Roman letters indicate observed variables, and Greek letters indicate parameters or latent variables. Bold-faced lowercase letters indicate vectors, and bold-faced uppercase letters indicate matrices. Normal-faced lowercase letters indicate fixed values, and normal-faced uppercase letters indicate random variables. These notations are also used in the subscript of a vector. For example, $\pmb {y}_{p,t}$ indicates a fixed response pattern $\pmb {y}$ for subject *p* on fixed measurement occasion *t*; $\pmb {y}_{P,t}$ indicates the response vector of a random subject *P* at fixed measurement occasion *t*; and $\pmb {y}_{p,T}$ indicates the response vector of a fixed subject *p* at a random measurement occasion *T*.

### Estimation

Suppose *n* cases (people or time points in this paper) are measured on $n_z$ variables, with $\pmb {z}_c$ denoting the response vector of case *c*, which is the *c*th row of a data matrix $\pmb {Z}$. The vector $\pmb {z}_C$ will be assumed normally distributed:

$$\begin{aligned} \pmb {z}_C \sim N(\pmb {\mu }, \pmb {\Sigma }), \end{aligned}$$

in which $\pmb {\mu }$ represents a mean vector and $\pmb {\Sigma }$ represents a variance–covariance matrix.^4^ Let $\bar{\pmb {z}}$ represent the sample means:

$$\begin{aligned} \bar{\pmb {z}} = \frac{1}{n} \sum _{c=1}^{n} \pmb {z}_c. \end{aligned}$$

Furthermore, let $\bar{\pmb {S}}$ represent the sample variance–covariance matrix:

$$\begin{aligned} \bar{\pmb {S}} = \frac{1}{n} \sum _{c=1}^{n} (\pmb {z}_c - \bar{\pmb {z}} ) (\pmb {z}_c - \bar{\pmb {z}} )^{\top }. \end{aligned}$$

Using these summary statistics and assuming multivariate normality, we can estimate parameters by minimizing the following fit function:

1 $$\begin{aligned} F_{\mathrm {ML}} = \mathrm {trace}\left( \pmb {S} \pmb {\Sigma }^{-1}\right) + (\bar{\pmb {z}} - \pmb {\mu })^\top \pmb {\Sigma }^{-1}(\bar{\pmb {z}} - \pmb {\mu }) - \ln |\pmb {\Sigma }^{-1}|, \end{aligned}$$

which is proportional to $-2/n$ times the log-likelihood of the data. *Full information maximum likelihood estimation* (FIML) can be used when data are missing. To this end, the data can be subdivided in subsets of data that have the same missingness patterns. The FIML fit function to be minimized takes the following form:

2 $$\begin{aligned} F_{\mathrm {FIML}} = \frac{1}{n} \sum _{i} n_i \left( \mathrm {trace}\left( \pmb {S}_i \pmb {\Sigma }_i^{-1} \right) + \left( \bar{\pmb {z}_i} - \pmb {\mu }_i \right) ^{\top } \pmb {\Sigma }_i^{-1} \left( \bar{\pmb {z}_i} - \pmb {\mu }_i \right) - \ln |\pmb {\Sigma }_i^{-1} | \right) , \end{aligned}$$

in which $n_i$ represents the sample size of subset *i*; $\pmb {S}_i$ is the sample variance–covariance matrix of subset *i* (note, $\pmb {S}_i = \pmb {O}$ if $n_i = 1$); $\bar{\pmb {z}_i}$ conotes the sample means of subset *i* (note, it is the same as the observed score if $n_i = 1$); $\pmb {\Sigma }_i$ is a subset of $\pmb {\Sigma }$ that only contains elements of observed data in subset *i*; and $\pmb {\mu }_i$ is a subset of $\pmb {\mu }$ that only contains elements of observed data in subset *i*.

### Factor Model

Let $\pmb {\eta }_{p,t}$ indicate a length $n_\eta$ vector of variables of interest at time point *t* for subject *p*. If $\pmb {\eta }$ is not observed, it may be assumed that $\pmb {\eta }$ linearly causes observed indicators in a length $n_y$ vector $\pmb {y}$ according to a linear factor model:

3 $$\begin{aligned} \pmb {y}_{p,t} = \pmb {\tau } + \pmb {\Lambda } \pmb {\eta }_{p,t} + \pmb {\varepsilon }_{p,t}, \end{aligned}$$

in which $\pmb {\tau }$ is a general intercept vector; $\pmb {\Lambda }$ is a matrix of factor loadings; and $\pmb {\varepsilon }_{p,t}$ is a vector of residuals. Assume that the residuals, denoting measurement error or nuisance variables, have mean $\pmb {0}$ across people but not necessarily across time:

$$\begin{aligned} \mathcal {E}\left( \pmb {\varepsilon }_{P,t}\right)&= \pmb {0} \quad \quad \,\, \text {No general bias across people.} \\ \mathcal {E}\left( \pmb {\varepsilon }_{p,T}\right)&= \pmb {\mu }_p^{(\pmb {\varepsilon })} \quad \hbox {Subject }p\hbox { may respond consistently biased}. \end{aligned}$$

It is possible, without loss of information, to regard any of the above variable vectors as composites of the mean of subject *p*, denoted with $\pmb {\mu }_p$, and deviation from that mean in measurement *t*, denoted with $\pmb {\xi }_{p,t}$:

$$\begin{aligned} \pmb {y}_{p,t}&= \pmb {\mu }^{(\pmb {y})}_{p} + \pmb {\xi }^{(\pmb {y})}_{p,t}&\text {Observed scores are composed of a mean plus deviation.}\\ \pmb {\eta }_{p,t}&= \pmb {\mu }^{(\pmb {\eta })}_{p} + \pmb {\xi }^{(\pmb {\eta })}_{p,t}&\text {Latent variables are composed of a mean plus deviation.} \\ \pmb {\varepsilon }_{p,t}&= \pmb {\mu }^{(\pmb {\varepsilon })}_{p} + \pmb {\xi }^{(\pmb {\varepsilon })}_{p,t}&\text {Residuals are composed of general bias plus time-specific bias}, \end{aligned}$$

with the expected value of all $\pmb {\xi }$ vectors concerning either random people or random measurement occasions set to equal zero and no assumed temporal dependency between the residual deviations. It is then possible to formulate the measurement model of Eq. (3) in a within-subject and a between-subject part:

4 $$\begin{aligned} \pmb {\mu }^{(\pmb {y})}_{p}&= \pmb {\tau } + \pmb {\Lambda } \pmb {\mu }^{(\pmb {\eta })}_{p} + \pmb {\mu }^{(\pmb {\varepsilon })}_{p}&\text {Between-subject measurement model stable over time.} \nonumber \\ \pmb {\xi }^{(\pmb {y})}_{p,t}&= \pmb {\Lambda } \pmb {\xi }^{(\pmb {\eta })}_{p,t} + \pmb {\xi }^{(\pmb {\varepsilon })}_{p,t}&\text {Within-subject measurement model varying over time.} \end{aligned}$$

Note, the lack of subscripts on $\pmb {\Lambda }$ and $\pmb {\tau }$ indicates an assumption of measurement invariance across subjects and time (Adolf, Schuurman, Borkenau, Borsboom, & Dolan, 2014) and across within- and between-subject models.^5^ Because of the assumption of measurement invariance across subjects in $\pmb {\tau }$, no intercept is included in the within-subject measurement model.

### Network Models

#### The Gaussian Graphical Model

The GGM can be formed as a model for a variance–covariance matrix $\pmb {\Sigma }$ using the following notation (Epskamp, Rhemtulla, & Borsboom, 2017):

5 $$\begin{aligned} \pmb {\Sigma } = \pmb {\Delta } \left( \pmb {I} - \pmb {\Omega } \right) ^{-1} \pmb {\Delta }. \end{aligned}$$

In this expression, $\pmb {\Delta }$ is a diagonal scaling matrix that controls the variances, and $\pmb {\Omega }$ is a square symmetrical model matrix with zeroes on the diagonal and partial correlation coefficients on the off diagonal. Note, the expression above is identical to inverting $\pmb {\Sigma }$, standardizing the result, and multiplying all off-diagonal elements by $-1$. The sparsity (zeroes) in off-diagonal elements of $\pmb {\Omega }$ will equal the sparsity in the precision matrix $\pmb {\Sigma }^{-1}$, which can equivalently be modeled to obtain a GGM. However, the expression above has some benefits, namely in that the sign of the obtained parameters is in line with the interpretation and that the expression allows users to constrain partial correlations equally across groups while allowing the scale to vary freely (Kan, van der Maas, & Levine, 2019).

#### The Graphical Vector-Autoregression Model

The GVAR model utilizes two network structures to extend the GGM when observations are temporally dependent: the *temporal network* and the *contemporaneous network*. Assuming stationarity in all parameters over time, the model can be written as a regression on the previous time point. The typical formation for observed variables takes the following form (ignoring a subscript *p* for subject):

$$\begin{aligned} \pmb {y}_{t} = \pmb {\mu } + \pmb {B} \left( \pmb {y}_{t - 1} - \pmb {\mu }\right) + \pmb {\zeta }_{t}, \quad \pmb {\zeta }_{T} \sim N\left( \pmb {0}, \pmb {\Sigma }^{(\pmb {\zeta })}\right) , \end{aligned}$$

in which $\pmb {\zeta }_{t}$ represents a vector of normally distributed innovations. Equivalently, the model can be written in terms of a conditional normal distribution:

$$\begin{aligned} \pmb {y}_{T} \mid \pmb {y}_{T-1} = \pmb {y}_{t-1} \sim N( \pmb {\mu } + \pmb {B} \left( \pmb {y}_{t - 1} - \pmb {\mu }\right) , \pmb {\Sigma }^{(\pmb {\zeta })}). \end{aligned}$$

The matrix $\pmb {B}$ encodes *temporal* within-subject effects, and its transpose is typically used to display a personalized weighted directed network of temporal effects (Bringmann et al., 2013). The matrix can also be standardized to *partial directed correlations* (Wild et al., 2010). The matrix $\pmb {\Sigma }^{(\pmb {\zeta })}$ encodes within-subject *contemporaneous effects*—associations between variables in the same measurement occasion after taking temporal effects into account—and can be used to obtain a personalized undirected network structure with a GGM (Epskamp, Van Borkulo, et al., 2018) using Expression (5). The assumption of stationarity can further be used to obtain expressions for the stationary variance–covariance structure as well as the lag-*k* variance–covariance structure, as depicted below. Finally, the mean structure itself may vary across subjects, which could be used to form a GGM on the between-subject level: the *between-subject* network.

## Time-Series Data: The *ts-lvgvar* Model

The *ts-lvgvar* concerns a fixed subject *p* and takes measurement *T* as random. Assuming multivariate normality for all variables, the following may be formulated:

$$\begin{aligned} \pmb {y}_{p,T}&\sim N\left( \pmb {\mu }_p^{(\pmb {y})}, \pmb {\Sigma }_{p,0}^{(\pmb {y})} \right)&\text {Within-subject distribution of observed variables.} \\ \pmb {\eta }_{p,T}&\sim N\left( \pmb {\mu }_p^{(\pmb {\eta })}, \pmb {\Sigma }_{p,0}^{(\pmb {\eta })} \right)&\text {Within-subject distribution of latent variables.} \end{aligned}$$

More generally, the within-subject lag-*k* covariances can be defined as:

$$\begin{aligned} \mathrm {cov}\left( \pmb {y}_{p,T+k}, \pmb {y}_{p,T} \right)&= \pmb {\Sigma }_{p,k}^{(\pmb {y})} \quad \hbox { Observed variables within-subject lag-}k\hbox { covariances.} \\ \mathrm {cov}\left( \pmb {\eta }_{p,T+k}, \pmb {\eta }_{p,T} \right)&= \pmb {\Sigma }_{p,k}^{(\pmb {\eta })} \quad \hbox { Latent variables within-subject lag-}k\hbox { covariances,} \end{aligned}$$

with the superscript indicating the variable of interest. As seen in Eq. (4), the expected mean vector $\pmb {\mu }^{(\pmb {y})}_{p}$ is a composite of the intercept, the latent means, and the systematic bias in subject *p*. Because $\pmb {\mu }^{(\pmb {\varepsilon })}_{p}$ has the same length as $\pmb {y}^{(\pmb {\varepsilon })}_{p}$, it is not possible to estimate this bias from time-series data of a single subject, or even from an analysis with a few subjects, even when equality constraints are placed on the intercepts and factor-loading structure (because the latent means may vary, which may be the topic of interest). Therefore, an identifying assumption $\pmb {\mu }^{(\pmb {\varepsilon })}_{p} = \pmb {0}$ is required in the *ts-lvgvar* setting. Note, any within-subject variation is due to variations in deviations from the mean, which is denoted here with $\pmb {\xi }$. As a consequence, the within-subject mean structure takes the following form:

$$\begin{aligned} \pmb {\mu }^{(\pmb {y})}_{p} = \pmb {\tau } + \pmb {\Lambda } \pmb {\mu }^{(\pmb {\eta })}_{p} \quad \text {Stationary within-subject means}, \end{aligned}$$

and the variance–covariance structure takes the following form:6This matrix captures *within-subject variation*, with *k* indicating the lag, which may differ person to person.

The relationships between the latent variables may be modeled as a lag-1 GVAR. Assuming stationarity, the structural model then becomes:

$$\begin{aligned} \mathrm {vec}\left( \pmb {\Sigma }^{(\pmb {\eta })}_{p, 0}\right)&= \left( \pmb {I} \otimes \pmb {I} - \pmb {B}_p \otimes \pmb {B}_p \right) ^{-1} \mathrm {vec}\left( \pmb {\Sigma }^{(\pmb {\zeta })}_{p,0}\right) \quad \text {Stationary latent variation.}\\ \pmb {\Sigma }^{(\pmb {\eta })}_{p, k}&= \pmb {B}_p \pmb {\Sigma }^{(\pmb {\eta })}_{p, k-1} \quad \hbox { Stationary lag-}k \hbox { covariances between latent variables}. \end{aligned}$$

The innovation variance–covariance matrix can further be modeled as a GGM to obtain a latent contemporaneous network:

$$\begin{aligned} \pmb {\Sigma }^{(\pmb {\zeta })}_p&= \pmb {\Delta }^{(\pmb {\zeta })}_p \left( \pmb {I} - \pmb {\Omega }^{(\pmb {\zeta })}_p \right) ^{-1} \pmb {\Delta }^{(\pmb {\zeta })}_p . \end{aligned}$$

This leads to two network structures: a temporal network modeled with $\pmb {B}_p$, and a contemporaneous network modeled with $\pmb {\Omega }^{(\pmb {\zeta })}_p$. Typical SEM identifying constraints are needed to make the model discernible, such as having nonnegative degrees of freedom, placing $\pmb {\mu }^{(\pmb {\eta })}_{p} = \pmb {0}$ in a single-subject setting, and constraining either the first factor loadings in $\pmb {\Lambda }$ or the diagonal elements of $\pmb {\Delta }^{(\pmb {\zeta })}_p$ to one.

### Estimation

With the exception of modeling contemporaneous relationships as a GGM, the *ts-lvgvar* takes the form of a dynamic factor model (Molenaar, 1985, 2017), which is often estimated by using a *Toeplitz matrix* (Hamaker, Dolan, & Molenaar, 2002). Let $$\pmb {z}_{t}^{\top } = \begin{bmatrix}\pmb {y}_{p,t}^{\top }&\pmb {y}_{p,t+1}^{\top } \end{bmatrix}$$ represent a pair of consecutive responses, and let $\pmb {\Sigma } = \mathrm {var}(\pmb {z}_T)$. It follows that $\pmb {\Sigma }$ will take the form of a Toeplitz matrix:

$$\begin{aligned} \pmb {\Sigma } = \begin{bmatrix} \pmb {\Sigma }^{*} &{} \pmb {\Sigma }_{p,1}^{(\pmb {y})\top } \\ \pmb {\Sigma }_{p,1}^{(\pmb {y})} &{} \pmb {\Sigma }_{p,0}^{(\pmb {y})} \end{bmatrix}. \end{aligned}$$

It is evident that $\pmb {\Sigma }^{*} = \pmb {\Sigma }_{p,0}^{(\pmb {y})}$. However, modeling these blocks with the same parameters may lead to a false number of degrees of freedom, as the data will be copied to obtain these blocks. To this end, I keep the model for $\pmb {\Sigma }^{*}$ saturated with a unique set of parameters. The same holds for the mean structure:

$$\begin{aligned} \pmb {\mu } = \begin{bmatrix} \pmb {\mu }^{*} \\ \pmb {\mu }^{(\pmb {y})} \end{bmatrix}. \end{aligned}$$

Before fitting such a model, the data need to be structured in a certain way. In general, an augmented data matrix can be created by copying the data, shifting it by one row, and appending it to the former data matrix. However, it may be warranted to remove certain pairs (e.g., removing effects that occur over night). If someone is measured three times per day for three days, indicating that Day 1 consists of measurements 1, 2, 3; Day 2 of 4, 5, 6; and Day 3 of 7, 8, 9. The data may then be structured as follows:

7 $$\begin{aligned} \pmb {Z} = \begin{bmatrix} . &{}\quad \pmb {y}_{p,1}^{\top } \\ \pmb {y}_{p,1}^{\top } &{}\quad \pmb {y}_{p,2}^{\top } \\ \pmb {y}_{p,2}^{\top } &{}\quad \pmb {y}_{p,3}^{\top } \\ . &{}\quad \pmb {y}_{p,4}^{\top } \\ \pmb {y}_{p,4}^{\top } &{}\quad \pmb {y}_{p,5}^{\top } \\ \pmb {y}_{p,5}^{\top } &{}\quad \pmb {y}_{p,6}^{\top } \\ . &{}\quad \pmb {y}_{p,7}^{\top } \\ \pmb {y}_{p,7}^{\top } &{}\quad \pmb {y}_{p,8}^{\top } \\ \pmb {y}_{p,8}^{\top } &{}\quad \pmb {y}_{p,9}^{\top } \end{bmatrix}. \end{aligned}$$

The parameters may now be estimated by optimizing the FIML fit function (2). It should be noted that fitting such a time-series model to the Toeplitz matrix may not be the best course of action because the resulting fit function does not represent the true likelihood of the data. A different method would be to construct one large variance–covariance matrix for the entire vectorized dataset and subsequently evaluate the likelihood by treating this as a single observation (Ciraki, 2007, p. 90). Ciraki, (2007) provides derivatives for these (more) general dynamic SEMs—with the exception that the contemporaneous effects are modeled at the variance–covariance level rather than at the GGM level. This estimation method, however, is computationally very expensive and has therefore not been implemented in the *psychonetrics* package.

### Empirical Example

To exemplify the *ts-lvgvar*, I analyzed time-series data previously studied by Wichers, Groot, Psychosystems, Group, and Others (2016) and made public by Kossakowski, Groot, Haslbeck, Borsboom, and Wichers (2017). This dataset concerns a 57-year-old man with a history of major depression, who was measured over the course of 239 days through the experience sampling method (ESM) using a digital device with a touch screen. During the study, the participant reduced the intake of antidepressants and relapsed into a clinical depression (Wichers et al., 2016). Because of the assumed stationarity in the *ts-lvgvar*, I selected only the previously unstudied *post-assessment phase* (days 156 to 239) during which medication levels were no longer changed. Furthermore, I selected 28 items that were continuous and varied substantively across the sample. These items were designed to measure mood states, pathological symptoms, self-esteem, and physical condition. Before analyzing the data, I tested each individual item for a linear trend by regressing the item scores on the time variable and replaced scores for each significant ($\alpha = 0.05$) regression with the residuals.

***Measurement Model Formation*** Because the original study did not measure items according to a predefined measurement model, I set out to find a factor structure that leads to a limited number of indicators per factor. To this end, I first performed a parallel analysis on the data, which led me to retain five factors. Next, I performed an exploratory factor analysis (EFA) on the dataset using an *oblimin* rotation. In order to get a comparable number of indicators for each factor and to reduce the number of indicators to a number the software could handle (given decent computation speed), I determined the three indicators with the strongest absolute factor loadings for each of the five factors and discarded all other indicators. This led to the retention of 14 indicators^6^: “irritated,” “satisfied,” “lonely,” “anxious,” “enthusiastic,” “guilty,” “strong,” “restless,” “agitated,” “worry,” “ashamed,” “tired,” “headache,” and “sleepy,” which were all measured using 7-point scales. Finally, I formed the “confirmatory” model^7^ by making all factor loadings that had a stronger absolute value than 0.25 in the EFA solution free parameters and by constraining all other factor loadings to zero. Both the parallel analysis and EFA were performed using version 1.8.12 of the *psych* package for R (Revelle, 2018). Figure 2a visualizes the standardized factor loadings of the final estimated *ts-lvgvar* model (explained below), with the factors reordered to improve readability. The factors can roughly be interpreted as *positive affect* or *positive activation* (F1), *self-consciousness* (F2), *anxiety* (F3), *irritability* or *negative activation* (F3), *somatization* (F4), and *anxiety* (F5).

**Fig. 1:**
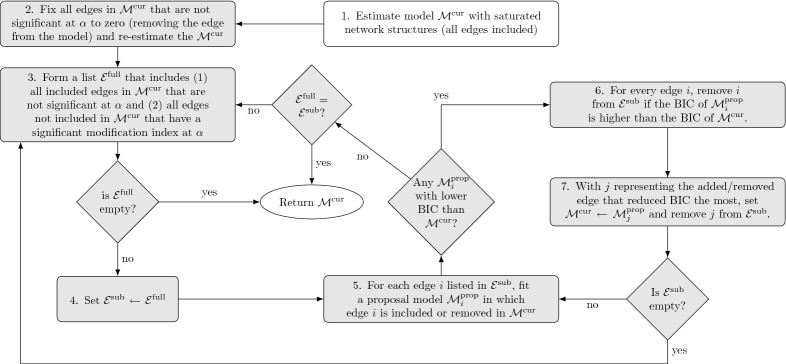
Model search strategy used for given $\alpha$ level (here, $\alpha = 0.01$ was used) in the *ts-lvgvar* and *panel-lvgvar* example analyses. For the *ts-lvgvar*, model selection is performed on the temporal network $\pmb {B}_p$ and the contemporaneous network $\pmb {\Omega }^{(\pmb {\zeta })}_p$, and for the *panel-lvgvar* model selection is performed on the temporal network $\pmb {B}_*$, the contemporaneous network $\underset{\mathrm {within}}{\pmb {\Omega }^{(\pmb {\zeta })}}$, and the between-subject network $\underset{\mathrm {between}}{\pmb {\Omega }^{(\pmb {\zeta })}}$. This algorithm has been implemented in the modelsearch function in the *psychonetrics* package.

***Model Estimation*** I fitted the *ts-lvgvar* model using version 0.4 of the *psychonetrics* package (code available in supplementary materials at https://osf.io/z5hbs/). The augmented data in the final model, as shown in Eq. (7), contained 486 rows of observations with $21.4\%$ missingness. Using FIML estimation, I first estimated a model in which the latent network structures (temporal and contemporaneous) were fully connected. The residual variances were estimated using a *Cholesky decomposition,*^8^ which ensured that all residual variances were nonnegative. Although exact fit was rejected, $\chi ^2(234) = 447.07, p < 0.001$, the model showed adequate close fit. The root-mean-square error of approximation (RMSEA) was 0.043 ($95\%\,\mathrm {CI}$ 0.037–0.049), and most incremental fit indices were acceptable ($\hbox {NFI} = 0.91$, $\hbox {PNFI} = 0.74$, $\hbox {TLI} = 0.95$, $\hbox {NNFI} = 0.95$, $\hbox {RFI} = 0.89$, $\hbox {IFI} = 0.96$, $\hbox {RNI} = 0.96$, $\hbox {CFI} = 0.96$). Next, I fixed to zero all edges from the contemporaneous and temporal network that were not significant at $\alpha = 0.01$, and refit the model. Finally, I performed stepwise model search to find a model with optimal Bayesian information criterion (BIC). The details of this algorithm are further explained in Fig. 1. This *pruned* model did not fit significantly worse than the original model, $\Delta \chi ^2(25) = 36.80, p = 0.06$, featured a lower AIC ($\Delta \mathrm {AIC} = 13.2$) and a lower BIC ($\Delta \mathrm {BIC} = 117.85$). The pruned model featured an acceptable RMSEA of 0.042 ($95\%\,\mathrm {CI}$ 0.036–0.048), with mostly acceptable incremental fit indices ($\hbox {NFI} = 0.91$, $\hbox {PNFI} = 0.82$, $\hbox {TLI} = 0.95$, $\hbox {NNFI} = 0.95$, $\hbox {RFI} = 0.90$, $\hbox {IFI} = 0.95$, $\hbox {RNI} = 0.95$, $\hbox {CFI} = 0.95$).

**Fig. 2:**
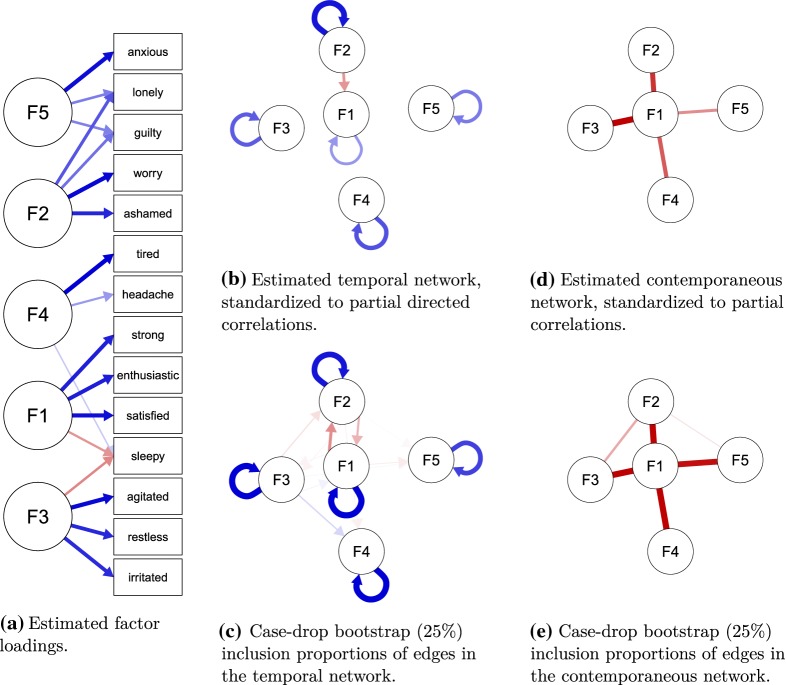
Results of the *ts-lvgvar* analysis of the post-assessment data from Kossakowski et al. (2017). Blue edges indicate positive effects, and ref edges indicate negative effects. The estimated within-subject latent network structures (**b** and **d**) were estimated together with the measurement model in **a**. Panels **c** and **e** show how often each edge was included in a 25% case-drop bootstrap (Color figure online).

Figure 2 shows the temporal effects, standardized to partial directed correlations (Wild et al., 2010), and the estimated contemporaneous partial correlation network. Table 1 shows the numeric estimates of these networks and the model-implied marginal contemporaneous correlations. In the estimated model, each of the five factors featured strong positive autoregressions, also termed *inertia*. At the temporal level, deviations in the second factor (self-consciousness) predicted lower levels of the first factor (positive activation) over time. At the contemporaneous level, the first factor played a central role: High levels of positive activation seemed to be uniquely associated with lower levels in all of the other four negative factors.

**Table 1:** Numeric results of the *ts-lvgvar* analysis of the post-assessment data from Kossakowski et al. (2017).

	F1	F2	F3	F4	F5
(a) *Estimated partial directed correlation*					
F1	0.22	–	–	–	–
F2	$-$ 0.22	0.50	–	–	–
F3	–	–	0.35	–	–
F4	–	–	–	0.37	–
F5	–	–	–	–	0.29
(b) *Estimated partial contemporaneous correlations* (*lower triangle*) *and model-implied marginal contemporaneous correlations* (*upper triangle*)					
F1	–	$-$ 0.60	$-$ 0.69	$-$ 0.52	$-$ 0.38
F2	$-$ 0.43	–	0.41	0.31	0.23
F3	$-$ 0.54	–	–	0.36	0.26
F4	$-$ 0.35	–	–	–	0.20
F5	$-$ 0.24	–	–	–	–

**Fig. 3:**
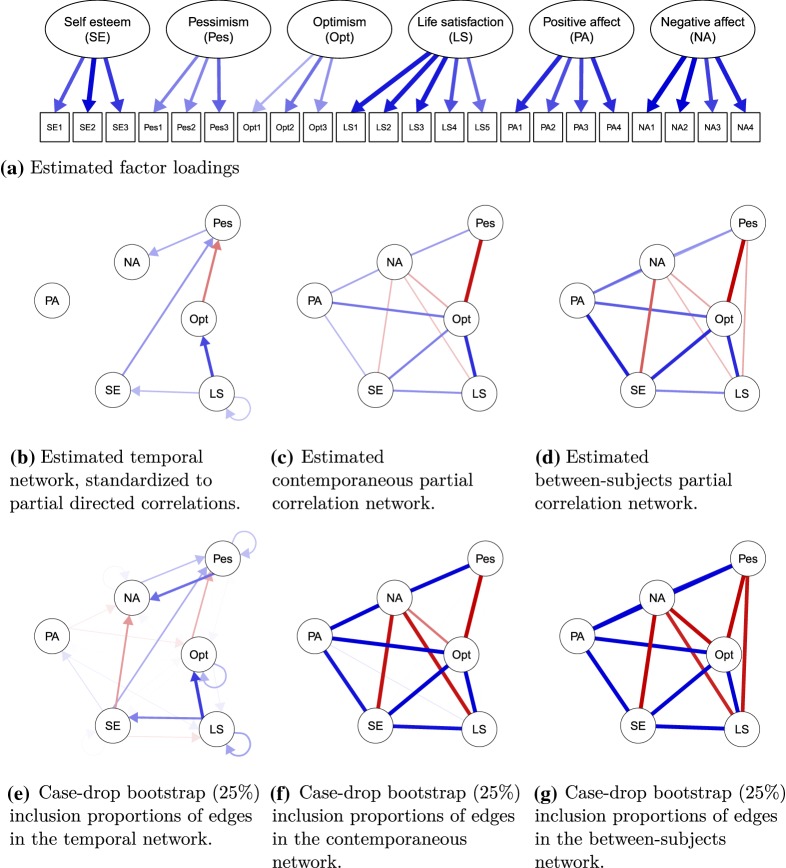
Results of the *panel-lvgvar* analysis of the LISS Core Study on personality. Blue edges indicate positive effects, and ref edges indicate negative effects. The estimated fixed-effect within-subject latent networks are shown in **b** and **c** and the estimated between-subject network in **d**. These are estimated jointly with the measurement model parameters represented in **a**. Panels **e**, **f** and **g** show the inclusion proportion of each edge in a 25% case-drop bootstrap (Color figure online).

***Bootstrap Results*** To assess the stability of the estimation algorithm, I performed 1000 *case-drop bootstraps* (Epskamp, Borsboom, & Fried, 2017) in which I dropped $25\%$ of the data and reestimated the model structure using the search strategy of Fig. 1. The $25\%$ rate was chosen in line with previous simulation studies on the use of case-drop bootstraps in cross-sectional networks, which suggests dropping a minimum of $25\%$ cases may be needed to assess stability of the network structure. To take temporal dependency into account, I dropped a block of data rather than individual rows from the dataset in each bootstrap sample, starting from a measurement occasion chosen at random. Figures 2e and 3e visualize the number of times that each signed edge would be included in the estimated model, and Table 2 shows the raw number of times each parameter was included (ignoring sign). These results indicate a high level of stability in the inertia parameters (included in 749 to 1000 bootstrap samples) as well as the contemporaneous edges (included in 945 to 1000 bootstrap samples). At the temporal level, it is noteworthy that the temporal edge from *F*2 to *F*1 was only included in 290 bootstrap samples, and a temporal edge in the reverse direction (*F*1 to *F*2) was included 444 times. Even though both edges could be included in principle, none of the bootstrap samples featured both edges simultaneously included, indicating that in 734 of the bootstrap samples one temporal edge of any direction was included between *F*1 and *F*2. In the original sample, models with both edges or only $F1 \rightarrow F2$ fitted only slightly worse than the model with only $F2 \rightarrow F1$ in both AIC ($\Delta \mathrm {AIC} = -1.16$ and $\Delta \mathrm {AIC} = -10.08$, respectively) and BIC ($\Delta \mathrm {BIC} = -5.35$ and $\Delta \mathrm {BIC} = -10.08$, respectively). Thus, there is some evidence that the relationship between *F*1 and *F*2 may be reciprocal. Noteworthy at the contemporaneous level is that some negative partial correlations were included in bootstrap samples where one would expect positive effects: negative relationships between *F*2 and *F*3 were included 333 times and negative relationships between *F*2 and *F*5 were included 110 times. These negative edges are noteworthy because these would be expected when *F*1 is a common effect of the other factors (Epskamp, Waldorp, et al., 2018). However, because these were only included sparingly, they cannot be interpreted substantively.

**Table 2:** The number of times each parameter was included in the case-drop bootstrap *ts-lvgvar* analysis of the post-assessment data from Kossakowski et al. (2017).

	F1	F2	F3	F4	F5
(a) *Number of times temporal effects were included*					
F1	**1000**	444	44	19	69
F2	**290**	**918**	44	56	23
F3	46	121	**1000**	157	32
F4	0	0	0	**1000**	0
F5	0	0	0	0	**749**
(b) *Number of times contemporaneous effects were included*					
F2	**998**				
F3	**1000**	333			
F4	**1000**	0	5		
F5	**945**	110	0	0	

Each replication (100 in total) was based on a 75% subsample of the original dataset. Bold-faced values indicate parameters that were included in the original analysis.

## Panel Data: The *panel-lvgvar* Model

The *panel-lvgvar* concerns the variation across random people in a few fixed measurement occasions $t, t+1,\ldots$ (i.e., the distributions over people).

$$\begin{aligned} \pmb {y}_{P,t}&\sim N\left( \pmb {\mu }_t^{(\pmb {y})}, \pmb {\Sigma }_t^{(\pmb {y})} \right)&\text {Cross-sectional distribution of observed variables.} \\ \pmb {\eta }_{P,t}&\sim N\left( \pmb {\mu }_t^{(\pmb {\eta })}, \pmb {\Sigma }_t^{(\pmb {\eta })} \right)&\text {Cross-sectional distribution of latent variables}, \end{aligned}$$

*Stationarity* is assumed in time, and therefore, it is assumed that these structures do not change over time. Hence, subscript *t* in the mean vectors and in the variance–covariance matrices above can be replaced with the symbol $\dagger$:

$$\begin{aligned} \pmb {\mu }_t^{(\ldots )} = \pmb {\mu }_{\dagger }^{(\ldots )}, \pmb {\Sigma }_t^{(\ldots )} = \pmb {\Sigma }_{\dagger }^{(\ldots )} \quad \forall _t \quad \text {Equal means and (co)variances for all time points.} \end{aligned}$$

From the measurement model in Eq. (4), the expected value becomes:

$$\begin{aligned} \pmb {\mu }_{\dagger }^{(\pmb {y})} = \pmb {\tau } + \pmb {\Lambda } \pmb {\mu }_{\dagger }^{(\pmb {\eta })} \quad \text {Expected cross-sectional observed scores}. \end{aligned}$$

As for any fixed time point *t*, $\mathrm {cov}\left( \pmb {\mu }^{(\pmb {y})}_{P}, \pmb {\xi }^{(\pmb {y})}_{P,t}\right) = \pmb {O}$ by definition, the lag-*k* variance–covariance structure can be derived as:

8 $$\begin{aligned} \pmb {\Sigma }_k^{(\pmb {y})} = \mathrm {var}\left( \pmb {\mu }^{(\pmb {y})}_{P}\right) + \mathrm {cov}\left( \pmb {\xi }^{(\pmb {y})}_{P,\dagger + k}, \pmb {\xi }^{(\pmb {y})}_{P,\dagger }\right) . \end{aligned}$$

Note that $\mathrm {var}\left( \pmb {\mu }^{(\pmb {y})}_{P}\right)$ relates to purely *between-subject variation*. The between-subject variance–covariance structure can therefore be defined as follows:

9 $$\begin{aligned} \underset{\mathrm {between}}{\pmb {\Sigma }^{(\pmb {\eta })}}&= \mathrm {var}\left( \pmb {\mu }^{(\pmb {\eta })}_{P}\right)&\text {between-subject latent (co)variances,} \nonumber \\ \underset{\mathrm {between}}{\pmb {\Sigma }^{(\pmb {\varepsilon })}}&= \mathrm {var}\left( \pmb {\mu }^{(\pmb {\varepsilon })}_{P}\right)&\text {between-subject residual (co)variances,} \nonumber \\ \underset{\mathrm {between}}{\pmb {\Sigma }^{(\pmb {y})}}&= \mathrm {var}\left( \pmb {\mu }^{(\pmb {y})}_{P}\right) = \pmb {\Lambda } \underset{\mathrm {between}}{\pmb {\Sigma }^{(\pmb {\eta })}} \pmb {\Lambda }^{\top } + \underset{\mathrm {between}}{\pmb {\Sigma }^{(\pmb {\varepsilon })}}&\text {between-subject (co)variances}. \end{aligned}$$

This matrix equals the first part of Eq. (8). As for the second part, by virtue of the law of total expectation, the fixed-effect latent structure takes the following form:

10 $$\begin{aligned} \underset{\mathrm {within}}{\pmb {\Sigma }_{*,k}^{(\pmb {y})}} = \mathcal {E}_P\left( \mathrm {cov}_T\left( \pmb {\xi }^{(\pmb {y})}_{P,T+k}, \pmb {\xi }^{(\pmb {y})}_{P,T} \mid P \right) \right) = \mathcal {E}_T\left( \mathrm {cov}_P\left( \pmb {\xi }^{(\pmb {y})}_{P,T+k}, \pmb {\xi }^{(\pmb {y})}_{P,T} \mid T \right) \right) = \mathrm {cov}_P\left( \pmb {\xi }^{(\pmb {y})}_{P,\dagger +k}, \pmb {\xi }^{(\pmb {y})}_{P,\dagger }\right) , \end{aligned}$$

in which subscripts *T* and *P* are added to the expectation and covariance operators to make clear with respect to which aspect the expectation or covariance is taken. From left to right, $\mathcal {E}_P\left( \mathrm {cov}_T\left( \pmb {\xi }^{(\pmb {y})}_{P,T+k}, \pmb {\xi }^{(\pmb {y})}_{P,T} \mid P \right) \right)$ indicates the *fixed-effect* structure of the *within-subject* deviations—the within-subject structure of the average subject. The next expression, $\mathcal {E}_T\left( \mathrm {cov}_P\left( \pmb {\xi }^{(\pmb {y})}_{P,T+k}, \pmb {\xi }^{(\pmb {y})}_{P,T} \mid T \right) \right)$ indicates the expected between-subject variation over all time points, which due to the assumption of stationarity reduces to the final part of the expression: $\mathrm {cov}_P\left( \pmb {\xi }^{(\pmb {y})}_{P,\dagger +k}, \pmb {\xi }^{(\pmb {y})}_{P,\dagger }\right)$, which is also present in Eq. (8). This indicates that under the assumption of *homogeneity*—every subject follows the same within-subject process—the second part of Eq. (8) will equal the within-subject variance–covariance structure for every subject. If homogeneity is violated, the structure can instead be interpreted as the within-subject variance–covariance structure of the average subject.

Equations (9) and (10) may be used to write (8) as follows:11which forms the basis for estimating the *panel-lvgvar* model. The within-subject part can further be modeled as a latent variable model using the fixed-effect variance–covariance matrices for the latent variables and residuals:

$$\begin{aligned} \underset{\mathrm {within}}{\pmb {\Sigma }_{*,k}^{(\pmb {y})}} = {\left\{ \begin{array}{ll} \pmb {\Lambda } \underset{\mathrm {within}}{\pmb {\Sigma }_{*,k}^{(\pmb {\eta })}} \pmb {\Lambda }^{\top } + \underset{\mathrm {within}}{\pmb {\Sigma }_{*,k}^{(\pmb {\varepsilon })}} &{}\text{ if } k = 0 \nonumber \\ \pmb {\Lambda } \underset{\mathrm {within}}{\pmb {\Sigma }_{*,k}^{(\pmb {\eta })}} \pmb {\Lambda }^{\top } &{} \end{array}\right. }. \end{aligned}$$

The structural model becomes:

$$\begin{aligned} \mathrm {vec}\left( \underset{\mathrm {within}}{\pmb {\Sigma }_{*,0}^{(\pmb {\eta })}}\right)&= \left( \pmb {I} \otimes \pmb {I} - \pmb {B}_* \otimes \pmb {B}_* \right) ^{-1} \mathrm {vec}\left( \underset{\mathrm {within}}{\pmb {\Sigma }_{*,0}^{(\pmb {\zeta })}}\right) , \\ \underset{\mathrm {within}}{\pmb {\Sigma }_{*,k}^{(\pmb {\eta })}}&= \pmb {B}_* \underset{\mathrm {within}}{\pmb {\Sigma }_{*,k-1}^{(\pmb {\eta })}}, \end{aligned}$$

in which $\pmb {B}_*$ represents a matrix of fixed-effect temporal effects and $\pmb {\Sigma }_{*,k }^{(\pmb {\zeta })}$ the lag-*k* fixed-effect contemporaneous variance–covariance structure. Finally, the two latent variance–covariance structures in the model could further be modeled as GGMs:

$$\begin{aligned} \underset{\mathrm {within}}{\pmb {\Sigma }_{*}^{(\pmb {\zeta })}}&= \underset{\mathrm {within}}{\pmb {\Delta }^{(\pmb {\zeta })}} \left( \pmb {I} - \underset{\mathrm {within}}{\pmb {\Omega }^{(\pmb {\zeta })}} \right) ^{-1} \underset{\mathrm {within}}{\pmb {\Delta }^{(\pmb {\zeta })}}&\text {Within-subject latent contemporaneous network}.\\ \underset{\mathrm {between}}{\pmb {\Sigma }^{(\pmb {\eta })}}&= \underset{\mathrm {between}}{\pmb {\Delta }^{(\pmb {\eta })}} \left( \pmb {I} - \underset{\mathrm {between}}{\pmb {\Omega }^{(\pmb {\eta })}} \right) ^{-1} \underset{\mathrm {between}}{\pmb {\Delta }^{(\pmb {\eta })}}&\text {Between-subject latent network}. \end{aligned}$$

This leads to three network structures: (1) the (fixed-effect) within-subject temporal network modeled with $\pmb {B}_*$, (2) the (fixed-effect) within-subject latent contemporaneous network modeled with $\underset{\mathrm {within}}{\pmb {\Omega }^{(\pmb {\zeta })}}$, and (3) the between-subject latent network modeled with $\underset{\mathrm {between}}{\pmb {\Omega }^{(\pmb {\eta })}}$.

### Estimation

Estimation of the *panel-lvgvar* in panel data with $n_t$ measurements can be done by forming the vector:

$$\begin{aligned} \pmb {z}_{p} = \begin{bmatrix} \pmb {y}_{p,1} \\ \pmb {y}_{p,2} \\ \vdots \\ \pmb {y}_{p,n_t} \end{bmatrix}, \end{aligned}$$

such that the mean structure repeats the stationary mean:

$$\begin{aligned} \pmb {\mu } = \begin{bmatrix} \pmb {\mu }^{(\pmb {y})} \\ \pmb {\mu }^{(\pmb {y})} \\ \vdots \\ \pmb {\mu }^{(\pmb {y})} \end{bmatrix}. \end{aligned}$$

The variance–covariance structure then takes the form of a block Toeplitz matrix:

$$\begin{aligned} \pmb {\Sigma } = \begin{bmatrix} \pmb {\Sigma }_{\dagger ,0}^{(\pmb {y})} &{} \pmb {\Sigma }_{\dagger ,1}^{(\pmb {y})\top } &{} \cdots &{} \pmb {\Sigma }_{\dagger ,n_t - 1}^{(\pmb {y})\top } \\ \pmb {\Sigma }_{\dagger ,1}^{(\pmb {y})} &{} \pmb {\Sigma }_{\dagger ,0}^{(\pmb {y})} &{} \cdots &{} \pmb {\Sigma }_{\dagger ,n_t - 2}^{(\pmb {y})\top } \\ \vdots &{} \vdots &{} \ddots &{} \vdots \\ \pmb {\Sigma }_{\dagger ,n_t - 1}^{(\pmb {y})} &{} \pmb {\Sigma }_{\dagger ,n_t - 2}^{(\pmb {y})} &{} \cdots &{} \pmb {\Sigma }_{\dagger ,0}^{(\pmb {y})}. \end{bmatrix}. \end{aligned}$$

The model can then be estimated by minimizing Eq. (1) or Eq. (2). When not all variables are observed at all waves (or entire waves of data are missing), elements in $\pmb {\mu }$ and rows and columns in $\pmb {\Sigma }$ corresponding to missing variables can be cut out, or FIML estimation can be used when data are treated as missing.

### Empirical Example

To exemplify the *panel-lvgvar*, I investigated a subset from the Longitudinal Internet Studies for the Social Sciences (LISS; Scherpenzeel & Das, 2010) panel administered by CentERdata (Tilburg University, The Netherlands). The LISS panel is a representative sample of Dutch individuals who participate in a yearly survey on several factors.^9^ To minimize the amount of missing data and to exemplify the usage of the method on as little as three waves of data and the usage of the method in waves with unequal time differences between consecutive waves, I used three of the waves from the LISS Core Study on personality: 2013, 2014, and 2017. Table 3 shows the items I selected to measure five factors: *self-esteem*, *pessimism*, *optimism*, *life satisfaction*, *positive affect*, and *negative affect*. Pessimism and optimism were assessed with the LOT-R scale (Carver, Scheier, & Segerstrom, 2010), and life satisfaction was assessed with the Satisfaction With Life scale (Diener, Emmons, Larsen, & Griffin, 1985). Because the current software does not handle more than about 30 variables, I used shorter scales than typical to assess self-esteem, positive affect, and negative affect. Self-esteem was assessed with a three-item scale accredited to “Radboud University Nijmegen, Netherlands” in the codebook, rather than the longer Rosenberg scale also present in the data (Rosenberg, 1965). Positive and negative affects were assessed with the PANAS scale (Watson, Clark, & Tellegen, 1988), which was shortened to four indicators per factor. To this end, I first fit *panel-lvgvar* models for positive and negative affects separately and retained the four items that featured the highest absolute factor loadings for both factors. Finally, to make the analysis fully reproducible and to exemplify the estimation of the *panel-lvgvar* from summary statistics, I only retained cases with no missing data. Thus, the final sample included 2, 998 cases administered three times on 22 items per wave. The variance–covariance matrix and mean vector of the data, as well as the code to reproduce the main analysis, are available in the supplementary.

**Table 3:** Measurement model for LISS data example.

Label	Item	Factor	Scale length
SE1	I am satisfied with the way I look	Self-esteem	7
SE2	I feel good about myself	Self-esteem	7
SE3	I have confidence in my capabilities	Self-esteem	7
Pes1	If something can go wrong for me, it will	Pessimism	5
Pes2	I hardly ever expect things to go my way	Pessimism	5
Pes3	I rarely count on good things happening to me	Pessimism	5
Opt1	In uncertain times, I usually expect the best	Optimism	5
Opt2	I’m always optimistic about my future	Optimism	5
Opt3	I’m always optimistic about my future	Optimism	5
LS1	In most ways my life is close to my ideal	Life satisfaction	7
LS2	The conditions of my life are excellent	Life satisfaction	7
LS3	I am satisfied with my life	Life satisfaction	7
LS4	So far I have gotten the important things I want in life	Life satisfaction	7
LS5	If I could live my life over, I would change almost nothing	Life satisfaction	7
PA1	Indicate to what extent you feel, right now, that is, at the present moment... determined	Positive affect	
PA2	... inspired	Positive affect	7
PA3	... active	Positive affect	7
PA4	... attentive	Positive affect	7
NA1	... nervous	Negative affect	7
NA2	... jittery	Negative affect	7
NA3	... irritable	Negative affect	7
NA4	... afraid	Negative affect	7

***Model Estimation*** I fit the *panel-lvgvar* model using version 0.4 of the *psychonetrics* package (code available in supplementary materials at https://osf.io/z5hbs/). First, I fit a model in which all edges were included in the temporal, contemporaneous, and between-subject networks and estimated all residual structures using a Cholesky decomposition. The model featured a numeric estimate of approximately zero for the between-subject residual variance for the item SE2.^10^ I fixed this parameter to zero (note, the within-subject residual was not constrained to zero, so the item did not become a perfect indicator as a result) and reestimated the model. Although exact fit was rejected, $\chi ^2(2118) = 5686.50, p < 0.001$, the model showed excellent close fit with an RMSEA of 0.024 ($95\%\,\mathrm {CI}$ 0.023–0.024) and good incremental fit ($\hbox {NFI} = 0.95$, $\hbox {PNFI} = 0.91$, $\hbox {TLI} = 0.97$, $\hbox {NNFI} = 0.97$, $\hbox {RFI} = 0.95$, $\hbox {IFI} = 0.97$, $\hbox {RNI} = 0.97$, $\hbox {CFI} = 0.97$). Next, I performed the same model search strategy used above in the *ts-lvgvar* example and further detailed in Fig. 1. While the pruned model fitted significantly worse than the original model, $\Delta \chi ^2(36) = 64.15, p < 0.01$, it was better in terms of AIC ($\Delta AIC = 7.8$) and BIC ($\Delta BIC = 224.05$). The pruned model also showed excellent fit, with an RMSEA of 0.024 ($95\%\,\mathrm {CI}$ 0.023–0.024) and good incremental fit ($\hbox {NFI} = 0.95$, $\hbox {PNFI} = 0.93$, $\hbox {TLI} = 0.97$, $\hbox {NNFI} = 0.97$, $\hbox {RFI} = 0.95$, $\hbox {IFI} = 0.97$, $\hbox {RNI} = 0.97$, $\hbox {CFI} = 0.97$).

**Table 4:** Numeric results of the *panel-lvgvar* analysis of the LISS Core Study on personality.

	SE	Pes	Opt	LS	PA	NA
(a) *Estimated partial directed correlation*						
SE	–	0.16	–	–	–	–
Pes	–	–	–	–	–	0.13
Opt	–	$-$ 0.20	–	–	–	–
LS	0.11	–	0.28	0.09	–	–
PA	–	–	–	–	–	–
NA	–	–	–	–	–	–
(b) *Estimated partial contemporaneous correlations* (*lower triangle*) *and model-implied marginal contemporaneous correlations* (*upper triangle*)						
SE	–	$-$ 0.18	0.37	0.32	0.18	$-$ 0.21
Pes	–	–	$-$ 0.46	$-$ 0.22	$-$ 0.10	0.26
Opt	0.20	$-$ 0.36	–	0.45	0.26	$-$ 0.28
LS	0.17	–	0.32	–	0.12	$-$ 0.23
PA	0.11	–	0.21	–	–	0.05
NA	$-$ 0.11	0.15	$-$ 0.13	$-$ 0.10	0.14	–
(c) *Estimated partial between-subject correlations* (*lower triangle*) *and model-implied marginal between-subject correlations* (*upper triangle*)						
SE	–	$-$ 0.47	0.72	0.61	0.52	$-$ 0.50
Pes	–	–	$-$ 0.64	$-$ 0.53	$-$ 0.18	0.47
Opt	0.33	$-$ 0.39	–	0.69	0.47	$-$ 0.52
LS	0.19	$-$ 0.13	0.33	–	0.33	$-$ 0.47
PA	0.34	0.14	0.25	–	–	$-$ 0.11
NA	$-$ 0.25	0.17	$-$ 0.14	$-$ 0.11	0.22	–

Figure 3 portrays the estimated factor loadings and network structures in the pruned model, and Table 4 shows the numeric estimates of the standardized network parameters. The temporal network mainly features pathways from life satisfaction to self-esteem and optimism, and subsequently to pessimism and negative affect. At the contemporaneous and between-subject levels, optimism seems to play a central role in the networks—being connected to all other variables in both networks with consistently strong edges. Most noteworthy in both the contemporaneous and between-subject networks are the rather strong *positive* edges between positive affect and negative affect. Table 4 shows that the model-implied correlations in these two levels are rather weak and also positive at the contemporaneous level ($r = 0.05$ at the contemporaneous level and $r = -0.11$ at the between-subject level). These weak marginal correlations are in line with previous literature (e.g., Tuccitto, Giacobbi Jr, & Leite, 2010), suggesting that the correlation between positive and negative affects might not be as strongly negative as expected and that the factors may be (near) orthogonal instead. At the partial correlation levels, these weak marginal correlations lead to stronger partial correlations of an unexpected sign because the correlation is weaker than can be expected due to the links between positive/negative affect and third variables, such as self-esteem and optimism. It may even be that these variables act as a common effect between positive and negative affects (De Ron, Fried, & Epskamp, 2019; Epskamp, Waldorp, et al., 2018), in which case a strong partial correlation of an unexpected sign may also be expected. To check whether the results were influenced by the subset of indicators chosen to assess positive affect and negative affect, I also estimated a *panel-lvgvar* model for only positive and negative affects using all indicators. These showed similarly weak marginal correlations ($r = 0.003$ at the contemporaneous level and $r = -0.056$ at the between-subject level).

***Bootstrap Results*** To assess the stability of the estimation algorithm, I again analyzed a 1000 case-drop bootstrap samples on the final *panel-lvgvar* model. In each of these samples, $25\%$ of the participants were dropped at random from the original sample, and *panel-lvgvar* models were estimated using the algorithm described in Fig. 1. The number of times each edge was included is visually shown in Fig. 3 and numerically shown in Table 5. Across all three networks, edges that were included in the original analyses were also likely to be included in the case-drop bootstrapped analyses, and mostly edges that were not included in the original analysis were also less likely to be included in the case-drop bootstrapped analyses. This indicated a high level of stability in the estimated network structures, in particular in the between-subject network. In the contemporaneous network, the edge between negative affect and optimism was estimated only 543 times and an extra edge between positive affect and life satisfaction was included 99 times. The temporal network featured lower levels of stability: several edges that were included in the original sample were included less than 400 times in the bootstrap samples, and some additional edges (most noteworthy self-esteem to negative affect, negative affect to pessimism, self-esteem to pessimism, and self-loops on pessimism and optimism) were included over 200 times. As such, there is some evidence that the temporal network is less sparse than portrayed in the original analysis.

**Table 5:** The number of times each parameter was included in the case-drop bootstrap *panel-lvgvar* analysis of the LISS Core Study on personality.

	SE	Pes	Opt	LS	PA	NA
(a) *Number of times temporal effects were included*						
SE	27	**324**	19	103	109	399
Pes	8	249	29	2	1	**578**
Opt	11	**317**	300	61	4	22
LS	**491**	5	**763**	**357**	1	2
PA	0	0	83	56	0	79
NA	0	264	4	0	0	48
(b) *Number of times contemporaneous effects were included*						
SE						
Pes	10					
Opt	**1000**	**1000**				
LS	**936**	2	**1000**			
PA	**935**	48	**1000**	99		
NA	**947**	**990**	**543**	**930**	**1000**	
(c) *Number of times between-subject effects were included*						
SE						
Pes	5					
Opt	**998**	**998**				
LS	**992**	**933**	**996**			
PA	**999**	**993**	**996**	0		
NA	**999**	**949**	**971**	**862**	**1000**	

Each replication (1000 in total) was based on a 75% subsample of the original dataset. Bold-faced values indicate parameters that were included in the original analysis.

## Simulation Studies

To assess the performance of model search strategies in *ts-lvgvar* and *panel-lvgvar*, I performed a two simulation studies by simulating new datasets using the parameters from the models shown in Figs. 2 and 3, respectively. I varied the number of cases (time points) between 50, 100, 250, 500, and 1000 for the *ts-lvgvar* simulation study and (subjects) between 500, 1000, 2500, 5000, and 10, 000 for the *panel-lvgvar* simulations. After simulating data, I estimated the model structure using the algorithm presented in Fig. 1 and two variants: a variant in which no model search is performed after the initial pruning step 2, and a variant in which the model search from step 3 onwards was replaced with a stepwise up model search strategy in which the edge with the strongest modification index was added to the model until BIC was no longer improved (implemented in the stepup function in *psychonetrics*). In addition, I tested four variants of step 2 Fig. 1: in addition to initially removing edges at an $\alpha$ level, I also removed edges in step 2 using Bonferroni (Bland & Altman, 1995), Holm (Holm, 1979), and false discovery rate (FDR; Benjamini & Hochberg, 1995) adjustments. Finally, I varied $\alpha$ between 0.01 and 0.05 in the entire algorithm. This led to 24 different estimation algorithms. Each condition was replicated 100 times, leading to 12, 000 simulated datasets in both the *ts-lvgvar* and *panel-lvgvar* simulation studies. In these simulations, I investigated the *success* rate of estimation. (Some estimations failed due to numerical optimization issues such as non-positive definite matrices.) In the successfully estimated models, I investigated the *sensitivity*, *specificity*, and the *correlation* between original (true in the simulation) and estimated parameters (Epskamp & Fried, 2018).

**Fig. 4:**
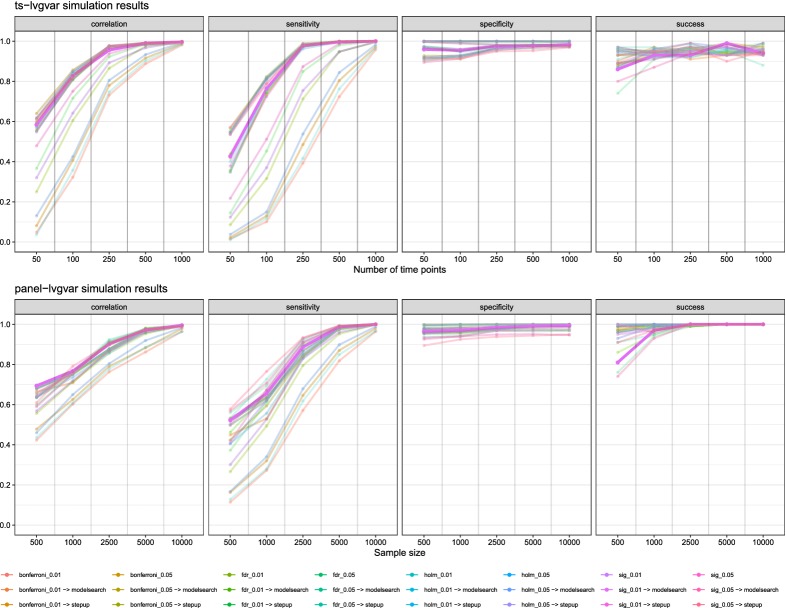
Simulation results for *ts-lvgvar* and *panel-lvgvar* model estimation algorithms implemented in *psychonetrics*. Data were generated under parameters from Fig. 2 for the *ts-lvgvar* simulations and under parameters from Fig. 3 for the *panel-lvgvar* simulations, and each condition was replicated 100 times. The lines display the average values over these replications. “Sensitivity” denotes the proportion of true edges that were also included in the estimated model, “specificity” denotes the proportion of true missing edges that were also not included in the estimated model, “correlation” denotes the Pearson correlation between true and estimated edge weights, and “success” denotes the proportion of models estimations that ran without errors. The thick highlighted line represents the algorithm used in the empirical examples in this paper.

Figure 4 shows the results of the simulation studies. All estimation algorithms were conservative at every sample size (high specificity), and increased in sensitivity and correlation as sample size increased. Generally, results using $\alpha = 0.05$ were a bit less conservative and a bit more sensitive than results using $\alpha = 0.01$. While very conservative, estimators that only used adjusted significance level in a pruning step without subsequent stepwise model search performed poor in terms of sensitivity, while not performing much better than estimators followed by stepwise model search strategies in terms of specificity. The estimator used in empirical examples above (*sig_0.01 -> modelsearch*) is highlighted in Fig. 4 and performed well on all measures. It should be noted, however, that the estimators using only significance pruning were much faster than the “stepup” estimator and even more faster than the “modelsearch” estimators: in the *ts-lvgvar*: estimating a model with extensive model search on average took 10.19 minutes in *ts-lvgvar* simulations and 35.15 minutes in *panel-lvgvar* simulations. Finally, the relatively large number of failed estimations is noteworthy, especially at lower sample sizes. Often, estimation failed due to numerical reasons leading to non-positive definite information matrices (e.g., due to non-positive definite variance–covariance structures). Future improvements to the *psychonetrics* package and other software methods implementing the *ts-lvgvar* and *panel-lvgvar* frameworks could improve computation speed of the model (e.g., by relying more on compiled programming languages) as well as the rate of successful model computations (e.g., by using more sophisticated starting values and/or more constrains on the parameter space).

## Ergodicity and Generalizability

The derivations in this paper can be used to show some important relationships between within- and between-subject effects as well as the correspondences between models in which time is random (time-series data) and models in which the subject is random (cross-sectional and panel designs). In particular, the expression in Eq. (11) has several important implications. First, it shows that the variance–covariance matrix of a cross-sectional set equates to a blend of within- and between-subject variances (Hamaker, 2012). As such, from a single cross-sectional study, it is impossible to determine whether two variables covary due to trait-level covariation between the means (between-subject) or due to state-level covariation between deviations from the mean (within-subject). Second, when investigating covariation over time between consecutive measurement occasions, nonzero covariances are not indicative per se of within-subject Granger causality (prediction over time) because nonzero covariances over time may also result due to between-subject effects. This is the core argument of Hamaker et al. (2015), who warn against a within-subject interpretation of the cross-lagged panel model unless a random intercept (here modeled with $\pmb {\mu }^{(\pmb {\eta })}_{p}$ and $\pmb {\mu }^{(\pmb {\varepsilon })}_{p}$) is included in the model.

Assuming *full homogeneity* in mean and variance–covariance structure—every subject has the same mean, and deviations in every subject from that mean have the same variance–covariance structure—Eq. (9) demonstrates that the between-subject covariance structure would become a matrix of zeroes (there are no differences in means), whereas Eq. (10) demonstrates that the within-subject fixed-effect structure would equal the within-subject variance–covariance structure for every subject. Now, when adding to this the assumption of stationarity, such that Eq. (11) is valid for any fixed measurement occasion *t*, the cross-sectional variance–covariance structure may equal the within-subject variance–covariance structure for every subject. When all variables are assumed normally distributed, such that no higher-order moments are present, these conditions then satisfy the assumption of *ergodicity* (Molenaar, 2004), which is frequently cited to be a requirement for generalizing cross-sectional studies to the within-subject level (e.g., Fisher, Medaglia, & Jeronimus, 2018).

Although the terms *within-subject* and *between-subject*—or alternatively within-person and between-persons—are commonly used in the literature and in this paper, they may lead to a faulty conclusion that between-subject effects necessarily do not occur within a person. The separation of within- and between-subject effects in Eq. (11) hinges on the assumption of *stationarity*, which is necessarily false in humans with a fixed life span. As such, the assumption may be better phrased as *local stationarity*, indicating that stationarity is assumed in the period of measurement only. Consider the effect between urbanicity and cannabis use (Isvoranu, Borsboom, van Os, & Guloksuz, 2016). If these two variables are measured on three consecutive days, then urbanicity is not likely to vary within subject, leading to a between-subject interpretation of the correlation between the two variables. However, if these two variables are instead measured once a year for three consecutive years, within-subject covariation may be found (e.g., when a person moves to a city in which cannabis is more readily available, that person may use more cannabis compared to his or her average). As such, there is some gray area among within- and between-subject effects: When measurements are too close together, $\underset{\mathrm {between}}{\pmb {\Sigma }^{(\pmb {\eta })}}$ may itself be interpreted as a mixture between slow within-subject effects and true between-subject effects. Likewise, if a particular questionnaire contained trait-level questions (e.g., “in the last year, did you smoke Cannabis”), $\underset{\mathrm {within}}{\pmb {\Sigma }_{*,k}^{(\pmb {\eta })}}$ may not accurately reflect valid daily fluctuations in the variables of interest and may be better interpreted as autocorrelated measurement error.

Note that in this paper the factor-loading structure does not differ between the within-subject and the between-subject levels. Typical multi-level software will allow for the specification of different factor-loading structures on both levels. However, when the factor-loading structure is not equal between the two levels, it is highly questionable whether the same factor is measured. For example, a goal of a study might be to obtain factor scores for a set of subjects at a given time point. If the within- and between-subject factor-loading structure is not the same, then these cannot be combined into factor scores, and only estimates of the means per subject can be obtained. The within-subject factor structure then reduces to correlated residuals over time. To be clear, it is *not* required that factors vary on both within- and between-subject levels: A factor-loading structure can easily be constrained equally over both levels, whereas a factor itself does not vary within one of the levels (e.g., genetic information). In principle, measurement invariance tests can be performed across people to test whether the factor structure is the same (Adolf et al., 2014).

## Discussion

I presented a general framework in which undirected network models can be used to model relationships between latent variables in both time-series data of a single subject (*ts-lvgvar*) and in panel data of many subjects measured on at least three occasions (*panel-lvgvar*). This presents a unification of network psychometrics, such that (dynamic) SEMs that include relationships over time at a latent level are combined with undirected network models. Two empirical examples, one using time-series data and one using panel data, as well as two large-scale simulation studies showed that network structures can reliably be estimated from data. These methods have been implemented in the software package *psychonetrics*, which is exemplified in the analysis code provided in the supplementary materials. I conclude the paper with a discussion on ergodicity and generalizability, discussing that although within-subject effects may in principle be separated from between-subject effects, the interpretation of these results rests on the intensity and intervals between measurements as well as the plausibility of the assumption of stationarity.

***Limitations*** Several limitations that are often raised for discrete time-series models (e.g., Epskamp, Waldorp, et al., 2018) may also be raised for the current work: (1) both the *ts-lvgvar* and the *panel-lvgvar* crucially rely on the assumptions of (local) *stationarity* and *normality*, which are unlikely to hold in practice. In principle, the assumption of stationarity is checked by assessing the model fit of the *panel-lvgvar* data (because it imposes unique constraints across all waves of measurement) but not by assessing the model fit of the *ts-lvgvar* when the data are fit to a lag-1 Toeplitz matrix as discussed in this paper. (2) Although methods exist to check for (multivariate) normality, it is not yet clear how to proceed when normality is violated because normality can be violated in many ways, and violations of normality have not yet been investigated in detail in the estimation of GGMs. To this end, it is vital to supplement any analyses with data-driven bootstrap methods, as done here, for example to check for the stability of the results by taking subsamples of the data. (3) The choice of lag interval and the duration of the study is not trivial. This choice will crucially impact the interpretation of the results. For example, although the provided empirical examples from both the *ts-lvgvar* and *panel-lvgvar* featured temporal networks, the interpretation of these networks is vastly different because the *ts-lvgvar* example concerned a lag interval of a few hours, whereas the *panel-lvgvar* example concerned the lag interval of one year. A further limitation is that only lag-1 relationships were used in the modeling framework, which is the minimum lag required to handle temporal dependency in the data.

In addition, the introduction of latent variables in these network models raises questions of measurement. In the empirical examples of the current paper, the measurement models used are far from confirmatory. The aim of this paper is to present methods for confirmatory fit and exploratory estimation of network structures at a latent level, while the measurement model is assumed known. To exemplify the method, however, I used a highly exploratory routine to obtain a measurement mode in the *ts-lvgvar* example. Other exploratory tools to assess the measurement structure could have been used, such as *exploratory graph analysis* (Golino & Epskamp, 2017), which is based on clustering in estimated GGMs, or *P-factor* technique (Zevon & Tellegen, 1982). Such methods may well lead to alternative factor structures and as a result different latent network structures. Furthermore, certain choices made in the *panel-lvgvar* model, such as the number of indicators for positive and negative affects, may have impacted the results. Ideally, these methods would be used on data that feature clear and previously studied measurement models (Flake & Fried, 2019).

Important to note is that while this paper extensively discussed issues pertaining to ergodicity and the ability to draw within-person conclusions from designs based on models in which the subject is random (cross-sectional and panel designs), the presented methods do not *solve* these issues. While I show that the temporal and contemporaneous structures from the *panel-lvgvar* directly relate to fixed-effect temporal and contemporaneous structures that may be obtained from time-series analysis, it may be stressed that such fixed effects can only say something about average effects in a population. Only when there is full homogeneity in within-person variance–covariance structures (an unlikely assumption) do these fixed-effect structures truly relate exactly to the within-person structures of every individual subject.

***Related Work*** The presented modeling framework is closely related to several other frameworks: (1) The *ts-lvgvar* reduces to a general GVAR model (Epskamp, Waldorp, et al., 2018) when all variables in the network are observed—the factor-loading matrix then reduces to an identity matrix and the residual variances are set to zero. In this case, the model reduces further to a general vector-autoregression model if the contemporaneous level is modeled as a variance–covariance matrix rather than a GGM. (2) When latent variables are used in the *ts-lvgvar*, but the contemporaneous level is not modeled using a GGM, the model reduces to a type of dynamic factor model (Molenaar, 1985). (3) The *panel-lvgvar* can be considered a multi-level GVAR model (Epskamp, Waldorp, et al., 2018) if all variables in the networks are observed without measurement error—with the exception that all network parameters are fixed (only intercepts are random). Extending the *panel-lvgvar* framework to include random effects on the network parameters will likely not be trivial, but may form an important direction of future research. (4) When the contemporaneous and between-subject effects of the *panel-lvgvar* are modeled as variance–covariance matrices and all variables in the networks are observed, the model reduces to a random intercept cross-lagged panel model (Hamaker et al., 2015), with the exception that stationarity is used to avoid estimating the variance–covariance structure of the first measurement. (5) When only cross-sectional data are used, the *panel-lvgvar* reduces to a latent network model (Epskamp, Rhemtulla, & Borsboom, 2017), after identifying all within-subject variance–covariance structures to be zero. Subsequently, when in cross-sectional data all variables in the network are assumed observed without measurement error, the model reduces to a standard GGM (Epskamp & Fried, 2018). (6) When the contemporaneous and between-subject levels of the *panel-lvgvar* are modeled using variance–covariance matrices, the model can be seen as a special case of general dynamic SEM models (Ciraki, 2007). When the data contain many persons and time series, it is in principle possible to obtain both fixed-effect and person-wise estimates (random effects), but this is beyond the scope of the current paper (e.g., Asparouhov, Hamaker, & Muthén, 2018; Epskamp, Waldorp, et al., 2018).

***Future Directions*** The current paper may motivate several lines of research: (1) The current formulations of the *ts-lvgvar* and *panel-lvgvar* only model contemporaneous relationships through undirected networks. It may instead be of interest to model these through directed relationships, as is typical in SEM and in structural vector-autoregression (Epskamp, Waldorp, et al., 2018; Gates & Molenaar, 2012; Gates, Molenaar, Hillary, Ram, & Rovine, 2010). Furthermore, both frameworks may be combined into a unified framework that allows for the estimation of mixed directional and undirectional models. (2) Multi-group models for both the *ts-lvgvar* (in which case a “group” would be a single subject) and *panel-lvgvar* may be investigated. The *psychonetrics* package allows for such multi-group analyses as well as testing for measurement invariance and homogeneity in network structures and mean structures. Future researchers could investigate the performance of such tests and provide detailed guidelines on how to assess measurement invariance and homogeneity in these models. (3) The *psychonetrics* package also allows for the modeling of *residual* structures as a GGM, which may be used to model residual network models as further discussed by Epskamp, Rhemtulla, and Borsboom (2017); however, this was also beyond the scope of this paper. (4) More detailed investigation on departures from normality in the *ts-lvgvar* and *panel-lvgvar* (or network models in general) may be investigated. For example, through the use of robust maximum likelihood estimation (Satorra & Bentler, 1994), threshold models (Muthén, 1984) or network models for binary data (Marsman et al., 2018), or mixed categorical and continuous data (Haslbeck & Waldorp, 2015). (5) While reported simulation studies investigated several different model search strategies, many more model search strategies could be envisioned which may perform better than the strategies used in this paper. Future research could therefore investigate alternative estimation procedures. Of note, regularization techniques are often used now in the estimation of psychological network models for both time-series and cross-sectional data (Epskamp, Waldorp, et al., 2018; Epskamp & Fried, 2018). These techniques have, however, also been criticized for poorer performance than unregularized estimation, especially in large samples (Williams & Rast, 2018), and in recent literature unregularized GGM estimation grew more common (e.g., Isvoranu et al., 2019). Promising lines of research on regularization involving latent variables (e.g., Chandrasekaran, Parrilo, & Willsky, 2012; Yuan, 2012; Jacobucci, Grimm, & McArdle, 2016) exist and may lead to regularized network estimation methods for the *ts-lvgvar* and *panel-lvgvar*.

***Conclusion*** The *ts-lvgvar* and *panel-lvgvar* frameworks extend the current toolbox of network psychometrics by (1) including latent variable models, (2) separating within- and between-subject variances, and (3) allowing for the estimation of within-subject temporal network models. Note that although this paper only contains examples with latent variables, the models can readily be used to estimate network models between observed variables by setting factor loadings to an identity matrix and setting all residual variances to zero. As such, the *panel-lvgvar* in particular offers a powerful new method for estimating fixed-effect temporal and contemporaneous networks from large sample panel data rather than intensive time-series data.

## Footnotes

1 Note: the network models discussed here include nodes that represent random variables. The term *network model* is also often used to describe models that feature random *edges* and/or the observed variables (e.g., social networks). For an extensive discussion on the differences between these kinds of models, see Epskamp, Fried, et al. (2018).

2 An alternative perspective is that the *lvgvar* is a combination of a dynamic factor model (Molenaar, 1985, 2017) with a GGM on the autoregression’s residuals.

3 http://psychonetrics.org.

4 Henceforth, superscripts on $\pmb {\mu }$ and $\pmb {\Sigma }$ will clarify the variable vector over which the means/variances are defined. For notational simplicity, I drop superscript $(\pmb {z})$.

5 In the *ts-lvgvar*, the model is estimated for an individual subject, so measurement invariance assumptions across subjects and across within- and between-subject models are not needed per se there for estimation. Furthermore, measurement invariance across subjects can be tested in the *ts-lvgvar* through multi-group models already implemented in *psychonetrics*, but this was beyond the scope of the current paper.

6 The item “lonely” featured in the top three indicators of two factors.

7 It should be noted that there is nothing “confirmatory” about this measurement model because it was obtained using a highly exploratory method.

8 Because the residual variance–covariance matrix is diagonal, its Cholesky decomposition is simply a diagonal matrix with the square root of the residual variances on the diagonal.

9 Detailed information and access to the data is available at www.lissdata.nl.

10 Further inspection revealed that this was a result of using the Cholesky decomposition; estimating the residual matrices using a variance–covariance matrix led to a Heywood case with a negative residual variance. These are hard to interpret, so I continued using a Cholesky decomposition and instead fixed this residual variance to zero.

## References

[CR1] Adolf J, Schuurman NK, Borkenau P, Borsboom D, Dolan CV (2014). Measurement invariance within and between individuals: A distinct problem in testing the equivalence of intra-and inter-individual model structures. Frontiers in Psychology.

[CR2] Asparouhov T, Hamaker EL, Muthén B (2018). Dynamic structural equation models. Structural Equation Modeling: A Multidisciplinary Journal.

[CR3] Benjamini Y, Hochberg Y (1995). Controlling the false discovery rate: A practical and powerful approach to multiple testing. Journal of the Royal statistical society: series B (Methodological).

[CR4] Bland JM, Altman DG (1995). Multiple significance tests: The Bonferroni method. BMJ.

[CR5] Borsboom D (2017). A network theory of mental disorders. World Psychiatry.

[CR6] Bos FM, Snippe E, de Vos S, Hartmann JA, Simons CJ, van der Krieke L, Wichers M (2017). Can we jump from cross-sectional to dynamic interpretations of networks implications for the network perspective in psychiatry. Psychotherapy and Psychosomatics.

[CR7] Bringmann LF, Eronen MI (2018). Don’t blame the model: Reconsidering the network approach to psychopathology. Psychological Review.

[CR8] Bringmann LF, Vissers N, Wichers M, Geschwind N, Kuppens P, Peeters F, Tuerlinckx F (2013). A network approach to psychopathology: New insights into clinical longitudinal data. PLoS ONE.

[CR9] Carver CS, Scheier MF, Segerstrom SC (2010). Optimism. Clinical Psychology Review.

[CR10] Chandrasekaran V, Parrilo PA, Willsky AS (2012). Latent variable graphical model selection via convex optimization (with discussion). The Annals of Statistics.

[CR11] Ciraki, D. (2007). *Dynamic structural equation models: Estimation and interference* (Unpublished doctoral dissertation). London School of Economics and Political Science (United Kingdom).

[CR12] Cramer AOJ, Waldorp LJ, van der Maas HLJ, Borsboom D (2010). Comorbidity: A network perspective. Behavioral and Brain Sciences.

[CR13] De Ron, J., Fried, E. I., & Epskamp, S. (2019). *Psychological networks in clinical populations: A tutorial on the consequences of Berkson’s bias*. 10.31234/osf.io/5t8zw. 10.1017/S0033291719003209 31796131

[CR14] Diener E, Emmons RA, Larsen RJ, Griffin S (1985). The satisfaction with life scale. Journal of Personality Assessment.

[CR15] Epskamp S, Borsboom D, Fried EI (2017). Estimating psychological networks and their accuracy: A tutorial paper. Behavior Research Methods.

[CR16] Epskamp S, Cramer AOJ, Waldrop LJ, Schmittmann VD, Borsboom D (2012). qgraph. Network visualizations of relationships in psychometric data. Journal of Statistical Software.

[CR17] Epskamp S, Fried EI (2018). A tutorial on regularized partial correlation networks. Psychological Methods.

[CR18] Epskamp S, Fried EI, van Borkulo CD, Robinaugh DJ, Marsman M, Dalege J, Cramer AOJ (2018). Investigating the utility of fixed-margin sampling in network psychometrics. Multivariate Behavioral Research.

[CR19] Epskamp S, Rhemtulla M, Borsboom D (2017). Generalized network pschometrics: Combining network and latent variable models. Psychometrika.

[CR20] Epskamp S, Van Borkulo C, Van Der Veen D, Servaas M, Isvoranu A-M, Riese H, Cramer A (2018). Personalized network modeling in psychopathology: The importance of contemporaneous and temporal connections. Clinical Psychological Science.

[CR21] Epskamp S, Waldorp LJ, Mõttus R, Borsboom D (2018). The Gaussian graphical model in cross-sectional and time-series data. Multivariate Behavioral Research.

[CR22] Fisher AJ, Medaglia JD, Jeronimus BF (2018). Lack of group-to-individual generalizability is a threat to human subjects research. Proceedings of the National Academy of Sciences.

[CR23] Flake, J. K., & Fried, E. I. (2019). *Measurement schmeasurement: Questionable measurement practices and how to avoid them*. 10.31234/osf.io/hs7wm.

[CR24] Fried EI, Cramer AO (2017). Moving forward: Challenges and directions for psychopathological network theory and methodology. Perspectives on Psychological Science.

[CR25] Fried EI, van Borkulo CD, Cramer AOJ, Boschloo L, Schoevers RA, Borsboom D (2017). Mental disorders as networks of problems: A review of recent insights. Social Psychiatry and Psychiatric Epidemiology.

[CR26] Gates KM, Molenaar PC (2012). Group search algorithm recovers effective connectivity maps for individuals in homogeneous and heterogeneous samples. NeuroImage.

[CR27] Gates KM, Molenaar PC, Hillary FG, Ram N, Rovine MJ (2010). Automatic search for fMRI connectivity mapping: An alternative to Granger causality testing using formal equivalences among SEM path modeling, VAR, and unified SEM. NeuroImage.

[CR28] Golino HF, Epskamp S (2017). Exploratory graph analysis: A new approach for estimating the number of dimensions in psychological research. PLoS ONE.

[CR29] Guyon H, Falissard B, Kop J-L (2017). Modeling psychological attributes in psychology—An epistemological discussion: Network analysis vs. latent variables. Frontiers in Psychology.

[CR30] Hamaker, E. L. (2012). Why researchers should think “within-person”: A paradigmatic rationale. In M. R. Mehl & T. S. Conner (Eds.), *Handbook of research methods for studying daily life* (pp. 43-61).

[CR31] Hamaker EL, Dolan CV, Molenaar PC (2002). On the nature of sem estimates of arma parameters. Structural Equation Modeling.

[CR32] Hamaker EL, Kuiper RM, Grasman RP (2015). A critique of the cross-lagged panel model. Psychological Methods.

[CR33] Haslbeck, J. M. B., & Waldorp, L. J. (2015). mgm: Structure estimation for time-varying mixed graphical models in high-dimensional data. arxiv:1510.06871.

[CR34] Holm S (1979). A simple sequentially rejective multiple test procedure. Scandinavian Journal of Statistics.

[CR35] Isvoranu AM, Borsboom D, van Os J, Guloksuz S (2016). A network approach to environmental impact in psychotic disorders: Brief theoretical framework. Schizophrenia Bulletin.

[CR36] Isvoranu, A.-M., Guloksuz, S., Epskamp, S., van Os, J., Borsboom, D., Investigators, G., et al. (2019). Toward incorporating genetic risk scores into symptom networks of psychosis. *Psychological Medicine*. 10.1017/S003329171900045X. 10.1017/S003329171900045X PMC7093319 30867074

[CR37] Jacobucci R, Grimm KJ, McArdle JJ (2016). Regularized structural equation modeling. Structural Equation Modeling: A Multidisciplinary Journal.

[CR38] Kan K-J, van der Maas HL, Levine SZ (2019). Extending psychometric network analysis: Empirical evidence against g in favor of mutualism?. Intelligence.

[CR39] Kossakowski JJ, Groot PC, Haslbeck JMB, Borsboom D, Wichers M (2017). Data from ‘critical slowing down as a personalized early warning signal for depression’. Journal of Open Psychology Data.

[CR40] Lauritzen SL (1996). Graphical models.

[CR41] Marsman M, Borsboom D, Kruis J, Epskamp S, van Bork R, Waldorp L, Marsman M (2018). An introduction to network psychometrics: Relating ising network models to item response theory models. Multivariate Behavioral Research.

[CR42] Molenaar PC (1985). A dynamic factor model for the analysis of multivariate time series. Psychometrika.

[CR43] Molenaar PC (2017). Equivalent dynamic models. Multivariate Behavioral Research.

[CR44] Molenaar PCM (2004). A manifesto on psychology as idiographic science: Bringing the person back into scientific psychology, this time forever. Measurement: Interdisciplinary Research & Perspective.

[CR45] Muthén B (1984). A general structural equation model with dichotomous, ordered categorical, and continuous latent variable indicators. Psychometrika.

[CR46] Revelle, W. (2019). *psych: Procedures for personality and psychological research*. Evanston, IL: Northwestern University. https://CRAN.R-project.org/package=psych (version 1.9.12).

[CR47] Rhemtulla, M., Van Bork, R., & Cramer, A. O. J. (2017). Cross-lagged network models. *Multivariate Behavioral Research*. Preprint from https://osf.io/r24q6/.

[CR48] Rosenberg M (1965). Rosenberg self-esteem scale (rse). Acceptance and Commitment Therapy. Measures Package.

[CR49] Satorra, A., & Bentler, P. M. (1994). Corrections to test statistics and standard errors in covariance structure analysis. In A. von Eye & C. C. Clogg (Eds.), *Latent variables analysis: Applications for developmental research* (pp. 399–419). Sage Publications, Inc.

[CR50] Scherpenzeel, A. C., & Das, M. (2010). True” longitudinal and probability-based internet panels: Evidence from the netherlands. In M. Das, P. Ester & L. Kaczmirek (Eds.), *Social and behavioral research and the internet: Advances in applied methods and research strategies* (pp. 77–104).

[CR51] Schuurman NK, Houtveen JH, Hamaker EL (2015). Incorporating measurement error in n = 1 psychological autoregressive modeling. Frontiers in Psychology.

[CR52] Tuccitto DE, Giacobbi PR, Leite WL (2010). The internal structure of positive and negative affect: A confirmatory factor analysis of the panas. Educational and Psychological Measurement.

[CR53] Watson D, Clark LA, Tellegen A (1988). Development and validation of brief measures of positive and negative affect: The panas scales. Journal of Personality and Social Psychology.

[CR54] Wichers M, Groot PC, Psychosystems ESM, Lenin EWS (2016). Critical slowing down as a personalized early warning signal for depression. Psychotherapy and Psychosomatics.

[CR55] Wild B, Eichler M, Friederich H-C, Hartmann M, Zipfel S, Herzog W (2010). A graphical vector autoregressive modelling approach to the analysis of electronic diary data. BMC Medical Research Methodology.

[CR56] Williams, D. R., & Rast, P. (2018). Back to the basics: Rethinking partial correlation network methodology. *British Journal of Mathematical and Statistical Psychology*. 10.1111/bmsp.12173. 10.1111/bmsp.12173 PMC8572131 31206621

[CR57] Yuan M (2012). Discussion: Latent variable graphical model selection via convex optimization. The Annals of Statistics.

[CR58] Zevon MA, Tellegen A (1982). The structure of mood change: An idiographic/nomothetic analysis. Journal of Personality and Social Psychology.

